# Supplementary material for: The Transitional Wettability on Bamboo-Leaf-like Hierarchical-Structured Si Surface Fabricated by Microgrinding
Source: Nanomaterials (Basel). 2022 Aug 22;12(16):2888. doi: 10.3390/nano12162888 (PMC9412647; doi:10.3390/nano12162888)
Supplement: Supplementary file 1 [file nanomaterials-12-02888-s001.zip › nanomaterials-1851818 supplementary.pdf]

nanomaterials

Supplementary Material

The Transitional Wettability on Bamboo-Leaf-Like Hierarchical-Structured Si Surface Fabricated by Microgrinding

Ping Li, Jinxin Wang, Jiale Huang and Jianhua Xiang *

School of Mechanical and Electric Engineering, Guangzhou University, Guangzhou 510006, China
* Correspondence: xiangjh@gzhu.edu.cn

Table S1. Particle size conversion chart.

Grinding wheel type	Particle size (μm)
SD60	250
SD120	124
SD320	44
SD600	21
SD1200	12
SD2000	6.5
SD3000	5

Movie S1: A water droplet is split up equally by two pieces of BLHS surface.

Movie S2: The water droplet on the modified BLHS surface is totally taken away by a BLHS surface.

Movie S3: The droplet is running with the needle without volume loss.

Movie S4: The droplet on the modified BLHS surface completely self-sucks into a hollow tubing rapidly without the help of external forces.

Movie S5: The water droplet rests on the BLHS surface without self-sucking phenomenon.

Movie S6: Comparation of droplet manipulation on the BLHS surface and modified BLHS surface.
